# Esophageal cancer mortality in China, 2008–2021: trends, disparities, and projections

**DOI:** 10.3389/fpubh.2026.1795219

**Published:** 2026-05-25

**Authors:** Mufei Wang, Haoyu Fang, Changlin Tang, Ming Du, Rui Zeng

**Affiliations:** 1 Department of Cardiothoracic Surgery, The Second Affiliated Hospital of Chongqing Medical University, Chongqing, China; 2 Department of Hepatobiliary Surgery, The Second Affiliated Hospital of Chongqing Medical University, Chongqing, China

**Keywords:** ARIMA, China, disease burden, esophageal cancer, mortality

## Abstract

**Background:**

Using national mortality surveillance data, this study aimed to characterize temporal trends and population disparities in esophageal cancer mortality risk and premature mortality burden in China from 2008 to 2021, thereby informing stratified prevention and control strategies.

**Methods:**

Esophageal cancer deaths were obtained from the Disease Surveillance Points (DSP) system of the Chinese Center for Disease Control and Prevention (2008–2021) and identified using ICD-10 code C15. We calculated the crude mortality rate (CMR), age-specific mortality rate, age-standardized mortality rate (ASMR), years of potential life lost (PYLL), and the PYLL rate (PYLLR), stratified by sex, age group, residence (urban/rural), and region (eastern/central/western China). ASMRs were estimated by direct standardization using the age structure of the 2010 Sixth National Population Census as the standard population. Joinpoint segmented regression was applied to estimate annual percent change (APC) and average annual percent change (AAPC). Group differences were assessed using chi-square tests in R. Age, period, and birth-cohort effects were examined with the US National Cancer Institute (NCI) online Age–Period–Cohort tool. An ARIMA model was fitted to the 2008–2021 ASMR time series to project ASMR trends for 2022–2036.

**Results:**

From 2008 to 2021, the ASMR of esophageal cancer in China declined from 14.39 to 7.62 per 100,000, indicating a sustained downward trend (AAPC = −4.82%). PYLLR also decreased from 0.77‰ to 0.41‰ (AAPC = −4.55%), suggesting concurrent reductions in mortality risk and premature mortality burden; however, their stratified and segmented patterns were not fully concordant. The burden was concentrated in older adults. Across the study period, ASMR and PYLLR were consistently higher in males than females and higher in rural than urban residents. Regional disparities across eastern, central, and western China narrowed over time, accompanied by shifts in relative ranking. ARIMA projections indicated that ASMR may continue to decline during 2022–2036, with increasing uncertainty over longer horizons.

**Conclusion:**

Esophageal cancer mortality risk and premature mortality burden in China decreased substantially between 2008 and 2021, yet persistent—and dynamically evolving—inequalities by sex, residence, and region remain. Future control efforts should maintain the overall downward trajectory while prioritizing high-burden populations and key areas, strengthening early detection and early treatment, and optimizing stratified resource allocation to reduce ongoing health disparities.

## 1 Introduction

Esophageal cancer (EC) is a highly lethal malignancy of the digestive tract and remains a major contributor to premature mortality worldwide (1), with esophageal squamous cell carcinoma (ESCC) and esophageal adenocarcinoma (EAC) as the two predominant histologic subtypes (2). Globally, esophageal cancer remains a major cause of cancer burden, with the age-standardized incidence and mortality rates were 6.65 and 6.25 per 100,000, respectively in 2021, and the burden was highest in East Asia (3–5). Its poor outcomes likely reflect multiple factors, including insidious early symptoms with consequent delays in diagnosis, advanced stage at presentation (6), aggressive tumor biology (7), and insufficient coverage of screening and early detection (8); accordingly, five-year survival remains low in many settings, posing a significant public health challenge (9). China bears one of the highest EC burdens worldwide, and ESCC accounts for the majority of cases (10).

Within China, the mortality burden of esophageal cancer is markedly heterogeneous across populations and places. Previous national analyses have consistently shown higher mortality in males and in rural residents, together with substantial geographic variation across provinces and counties (11, 12). High-burden areas have historically clustered in the central–northern inland belt, particularly around the Taihang Mountain and Huai River Basin regions, whereas mortality levels have generally been lower in more economically developed coastal areas (13). In addition, lower gross domestic product, and lower educational attainment are associated with higher esophageal cancer mortality (14). These persistent and evolving disparities underscore the need for a comprehensive reassessment of long-term mortality trends, premature mortality burden, subgroup inequalities, and future trajectories using a unified national surveillance framework.

To more comprehensively characterize temporal changes and inequalities in EC mortality in China, we used data from the Disease Surveillance Points (DSP) system to quantify and compare stratified patterns in mortality and premature-death burden. We applied segmented trend models (e.g., average annual percent change, AAPC) to quantify time trends and identify potential inflection points, and complemented these analyses with age–period–cohort modeling and time-series forecasting to evaluate structural shifts in risk and to project future trajectories. Although prior studies have described the epidemiology and temporal trends of EC in China, existing evidence varies in data sources, stratification granularity, and indicator selection: some reports lack systematic urban–rural or regional comparisons and few simultaneously examine both the level of mortality risk (e.g., age-standardized mortality rate, ASMR) and the burden of premature death (e.g., years of potential life lost rate, PYLLR) (14, 15). By applying a unified data source and stratification framework, our study provides a consolidated picture of EC mortality burden in China, highlights key inequalities and their evolution over time, and offers interpretable projections to inform optimization of comprehensive control efforts.

## 2 Materials and methods

### 2.1 Data source and study population

This population-based descriptive time-trend study used cause-specific mortality data from China’s DSP system maintained by the Chinese Center for Disease Control and Prevention, covering 2008–2021. EC deaths were identified using the International Classification of Diseases, 10th Revision (ICD-10) code C15. The DSP system underwent major expansion in 2013, increasing from 161 surveillance points during 2008–2012 to 605 points thereafter. Following this expansion, the system covered approximately 323.8 million individuals (24.3% of the Chinese population), compared with more than 73 million before 2013, and achieved national and provincial representativeness (16). The analysis included all 31 provincial-level administrative divisions in mainland China, excluding Hong Kong, Macao, and Taiwan.

### 2.2 Stratification variables and regional classification

To systematically characterize heterogeneity in EC mortality burden across population strata, analyses were stratified by sex, age group, place of residence, and geographic region. Sex was categorized as male or female. Age was grouped in accordance with the DSP scheme as follows: 0 years; 1–4 years; consecutive 5-year intervals thereafter (5–9, 10–14, …, 80–84); and an open-ended group of ≥85 years (85+). Residence was classified as urban or rural based on the DSP-recorded urban–rural attribute, using the National Bureau of Statistics rules, including the Code Compilation Rules for Statistical Administrative Divisions and the Urban–Rural Classification Rules. The classification method has not changed over time.

Because EC mortality is near zero before age 30 and exhibits substantial stochastic fluctuation that complicates visual comparison, figures assessing age-specific sex differences (e.g., mortality rate ratios) were restricted to age groups ≥30 years to enhance interpretability and visual stability. All other indicators and time-trend analyses were conducted using the full age range.

Geographic regions were defined according to the National Bureau of Statistics macro-regional classification, grouping the 31 provincial-level administrative divisions of mainland China (excluding Hong Kong, Macao, and Taiwan) into Eastern (Beijing, Tianjin, Hebei, Liaoning, Shanghai, Jiangsu, Zhejiang, Fujian, Shandong, Guangdong, Hainan); Central (Shanxi, Jilin, Heilongjiang, Anhui, Jiangxi, Henan, Hubei, Hunan); and Western (Inner Mongolia, Guangxi, Chongqing, Sichuan, Guizhou, Yunnan, Tibet, Shaanxi, Gansu, Qinghai, Ningxia, Xinjiang).

Standard population weights for age standardization were derived from the Sixth National Population Census of China (2010). Census-based age-specific population proportions were calculated using the same age grouping (0, 1–4, 5–9, …, 85+) and applied as weights when estimating age-standardized indicators, including ASMR.

### 2.3 Quality control

The DSP system compiles death reports from surveillance sites via a centralized online reporting platform. Causes of death are certified by qualified clinicians using official death certificates and coded according to ICD-10 standards. To reduce bias from under-ascertainment, underreporting rates derived from dedicated field surveys were applied to adjust EC mortality estimates using the standard correction formula: adjusted mortality rate = crude mortality rate / (1 − underreporting rate). Further details on the National Mortality Surveillance System workflow, Chinese Center for Disease Control and Prevention quality-control procedures, the proportion of garbage codes, and the corresponding redistribution methods have been described elsewhere (16, 17).

### 2.4 Statistical analysis

We used Excel to compute mortality and premature-mortality indicators for EC, including the crude mortality rate (CMR), age-specific mortality rate, ASMR, years of potential life lost (PYLL), and the PYLLR. CMR was defined as the number of EC deaths in a given year divided by the corresponding population, expressed per 100,000 population. The calculation of the ASMR is as follows:


$$\text{ASMR}=\frac{\sum nP_{x}*nM_{x}}{\sum nP_{x}}$$

In the formula, $nP_{x}$ denotes the age-specific population in the standard population, $nM_{x}$ denotes the age-specific mortality rate in the population to be standardized, n denotes the width of each age group, and x denotes the starting age of each age group. PYLL was used to quantify premature mortality burden using an upper reference age of 70 years; The calculation of the PYLL is as follows:


PYLL=ai·di

In the formula, di is the average age of the *i*-th age group; *ai* is the residual age, calculated as *ai* = 70 − *xi* + 0.5, with xi being the average age of the *i*-th age group. The addition of 0.5 is applied to eliminate the influence of nominal age counting. PYLLR standardized PYLL to the population size and was reported per 1,000 population (‰), with the denominator defined as the total population aged 1–70 years. Detailed formulas and calculation procedures are provided in Supplementary material 1.

We applied Joinpoint analysis to characterize changes in EC mortality burden in China from 2008 to 2021. Previous study reported that it can divide the longitudinal variations into different segments by piecewise regression and identify the segment trends with statistical significance (18). Annual trends in ASMR and PYLLR were modeled using the Joinpoint (version 5.4.0.0); the optimal number of joinpoints was selected via Monte Carlo permutation testing. For each segment, we estimated the APC and the overall AAPC, along with corresponding 95% confidence intervals (95% CIs). Trends were considered statistically significant when the 95% CI did not include zero. Mortality differences across population strata (sex, urban/rural residence, and eastern/central/western regions) were assessed in R using chi-square (*χ*^2^) tests, and trend comparability/parallelism tests were further conducted to evaluate whether AAPC trajectories differed between strata. The complete Joinpoint output tables are presented in Supplementary Figures 1, 2.

Age–period–cohort analysis is a commonly used epidemiological approach for characterizing age-, period-, and cohort-related patterns in health outcomes (19). Briefly, age effects reflect changes associated with aging, period effects capture influence of human factors on EC, such as diagnosis development, and cohort effects represent different exposures to risk factors among people of different birth years. In this study, we used the U.S. National Cancer Institute (NCI) online APC analysis tool within a Poisson regression framework, and parameter significance was assessed using Wald tests.

ARIMA is a commonly used time-series approach that combines autoregressive (20), differencing, and moving-average components and is denoted as ARIMA (p, d, q). ARIMA effectively captures trends in time series data by combining autoregressive (AR), differencing (I), and moving average (MA) components. To stabilize variance and ensure positive forecasts, models were fitted on the log scale using a Box–Cox transformation (*λ* = 0), and forecasts were back-transformed to the original scale with bias adjustment. Model identification and parameter estimation were performed using the auto.arima function in the R package forecast. Point forecasts and corresponding 95% prediction intervals were generated for each series. These forecasts should be interpreted as conditional extrapolations of historical time-series patterns rather than causal predictions. The ARIMA approach assumes that the underlying temporal structure remains broadly stable over the forecast horizon after differencing and transformation. Given the relatively short annual series and the widening prediction intervals over time, projections up to 2036 should be interpreted with caution. All ARIMA analyses and visualizations were conducted in R (v4.3.1).

All statistical tests were two-sided, with *p* < 0.05 considered statistically significant. Data cleaning, indicator calculations, and part of the figure visualization were performed in Microsoft Excel 2016. Segmented time-trend analyses were conducted using the Joinpoint (v5.4.0.0), while all other statistical analyses, result summarization, and visualizations were completed in R (v4.3.1). The DSP data used in this study were de-identified, aggregated surveillance data and contained no information that could be used to identify individuals; the study was conducted in accordance with the ethical principles of the Declaration of Helsinki (2013 revision).

## 3 Results

### 3.1 Spatial distribution and convergence in EC ASMR

**Figure 1:**
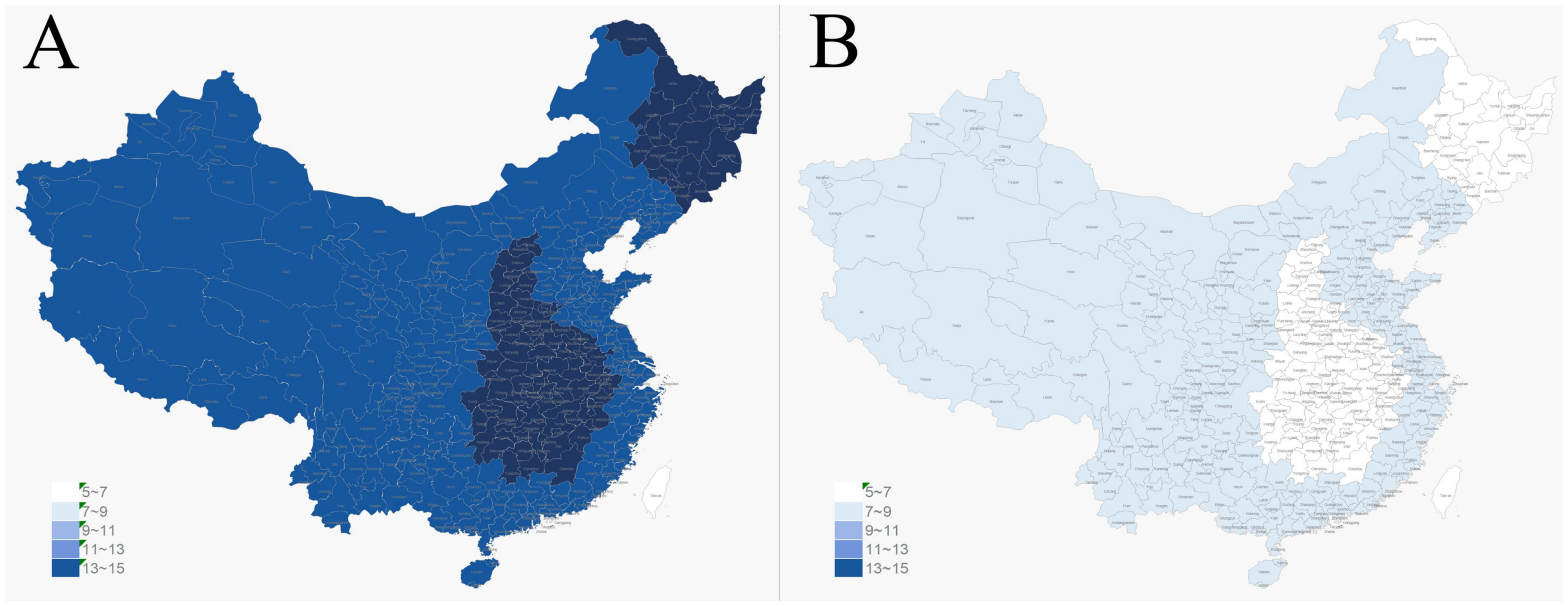
Spatial distribution of esophageal cancer mortality in mainland China. Age-standardized mortality rate (ASMR, per 100,000) in mainland China in 2008 **(A)** and 2021 **(B)**. Darker shading indicates higher ASMR.

Overall, the ASMR of EC in China showed a sustained decline from 2008 to 2021. In terms of spatial distribution, the mortality burden exhibited marked geographic heterogeneity (Figure 1). Figure 1 compares the spatial patterns of ASMR across regions in 2008 and 2021, with darker shading indicating higher ASMR levels. Compared with 2008, most areas appeared noticeably lighter in 2021, suggesting a widespread national decrease in EC ASMR.

At the macro-regional level, substantial reductions were observed across eastern, central, and western China over the study period. Notably, the geographic gap among eastern, central, and western China became smaller over time, suggesting an overall convergence of regional disparities (Figure 1).

### 3.2 EC mortality and changing trends

**Figure 2:**
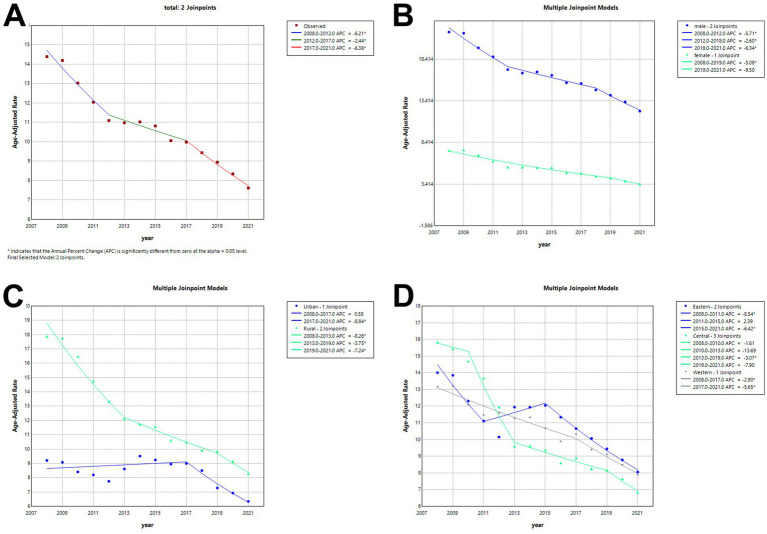
Joinpoint trends in ASMR, 2008–2021. Annual ASMR (per 100,000) in the overall population **(A)**, by sex **(B)**, by residence (urban/rural) **(C)**, and by region (eastern/central/western China) **(D)**. Points represent observed values, and solid lines represent fitted joinpoint segments. Segment-specific annual percent change (APC) estimates are shown in the inset; asterisks indicate APC values significantly different from zero.

From 2008 to 2021, mortality from EC in China declined overall (Table 1; Figure 2). The CMR decreased from 13.20 per 100,000 in 2008 to 11.70 per 100,000 in 2021, while the ASMR fell more markedly, from 14.39 to 7.62 per 100,000. Joinpoint regression confirmed a statistically significant downward trend in national ASMR across the study period (AAPC = −4.82, 95% CI: −6.04 to −3.60%) (Table 1; Figure 2A). The decline was non-linear, with a faster decrease during 2008–2012,a slower decline during 2012–2017, and a renewed acceleration during 2017–2021 (Figure 2A).

**Table 1:** CMR and ASMR of esophageal cancer from 2008 to 2021 in China (1/100,000).

Index		2008	2009	2010	2011	2012	2013	2014	2015	2016	2017	2018	2019	2020	2021	AAPC% (95%CI)	*t*	*p*
Male	CMR	18.86	18.55	17.25	17.28	17.19	18.14	18.66	18.23	18.22	18.24	18.09	17.75	17.47	17.28			
	ASMR	21.67	21.53	19.77	18.71	17.17	16.76	16.91	16.50	15.60	15.51	14.74	14.20	13.29	12.19	−4.44 (−5.44, −3.42)	−8.43	<0.01
Female	CMR	7.29	7.35	6.64	6.59	6.29	6.72	6.78	6.74	6.60	6.55	6.43	6.09	5.93	5.94			
	ASMR	7.45	7.52	6.88	6.17	5.45	5.47	5.40	5.40	4.80	4.73	4.38	4.19	3.79	3.42	−5.77 (−7.28, −4.24)	−7.23	<0.01
	*X* ^2^	1872.67	1801.05	1839.59	1838.75	1934.75	5913.69	6966.51	6756.12	7147.44	7415.99	7493.17	7832.74	7870.00	7357.02			
	*p*	<0.01	<0.01	<0.01	<0.01	<0.01	<0.01	<0.01	<0.01	<0.01	<0.01	<0.01	<0.01	<0.01	<0.01			
Urban areas	CMR	9.71	9.56	8.62	8.20	8.35	10.36	11.12	10.88	10.87	11.03	11.01	10.03	10.08	9.83			
	ASMR	9.20	9.07	8.40	8.19	7.75	8.62	9.52	9.25	8.96	9.00	8.50	7.34	6.93	6.35	−2.42 (−3.90, −0.91)	−3.14	<0.01
Rural areas	CMR	15.08	14.99	14.14	14.57	14.16	13.52	13.64	13.38	13.34	13.24	13.06	13.04	12.71	12.67			
	ASMR	17.87	17.75	16.46	14.75	13.33	12.13	11.75	11.59	10.62	10.49	9.93	9.92	9.14	8.30	−6.05 (−7.31, −4.76)	−9.01	<0.01
	*X* ^2^	368.21	387.91	469.78	625.10	527.50	385.15	272.51	280.59	286.23	237.86	207.47	469.89	372.48	415.44			
	*p*	<0.01	<0.01	<0.01	<0.01	<0.01	<0.01	<0.01	<0.01	<0.01	<0.01	<0.01	<0.01	<0.01	<0.01			
Eastern China	CMR	14.42	14.20	12.61	12.35	11.79	15.05	15.22	15.31	15.29	14.60	14.46	13.83	13.62	13.27			
	ASMR	14.11	13.84	12.30	11.11	10.15	11.94	11.94	12.05	11.34	10.66	10.07	9.52	8.79	8.06	−4.30 (−6.42, −2.14)	−3.86	<0.01
Central China	CMR	13.53	13.35	12.85	12.78	12.20	10.17	10.53	10.28	10.20	10.71	10.45	10.53	10.37	10.35			
	ASMR	15.83	15.42	14.69	13.67	11.94	9.61	9.62	9.38	8.60	8.90	8.25	8.23	7.65	6.84	−6.16 (−10.05, −0.09)	−2.94	<0.01
Western China	CMR	11.09	11.09	10.24	10.54	11.44	12.10	12.37	11.63	11.53	11.69	11.73	11.22	10.92	11.03			
	ASMR	13.15	13.20	12.09	11.47	11.59	11.28	11.34	10.68	9.90	10.33	10.00	9.19	8.50	7.90	−3.76 (−4.52, −2.98)	−9.39	<0.01
	*X* ^2^	102.89	90.87	79.94	52.65	5.67	815.97	823.93	1009.31	1083.56	655.20	683.11	512.71	521.06	393.15			
	*p*	<0.01	<0.01	<0.01	<0.01	0.06	<0.01	<0.01	<0.01	<0.01	<0.01	<0.01	<0.01	<0.01	<0.01			
Total	CMR	13.20	13.06	12.05	12.03	11.84	12.54	12.84	12.57	12.51	12.49	12.36	12.01	11.79	11.70			
	ASMR	14.39	14.19	13.02	12.04	11.09	10.99	11.03	10.82	10.07	9.99	9.44	9.02	8.34	7.62	−4.82 (−6.04, −3.60)	−7.56	<0.01

CMR, crude mortality rate; ASMR, age-standardized mortality rate; AAPC, average annual percent change; CI, confidence interval.

Sex-stratified analyses showed consistently higher mortality among males than females, with significant declines in ASMR for both sexes (Table 1; Figure 2B). Among males, ASMR decreased from 21.67 to 12.19 per 100,000, corresponding to an AAPC of −4.44% (95% CI: −5.44% to −3.42), whereas in females ASMR declined from 7.45 to 3.42 per 100,000, with an AAPC of −5.77% (95% CI: −7.28% to −4.24). The parallelism test indicated no significant difference in temporal trends between males and females (*p* = 0.1532) (Supplementary Figure 1).

Urban–rural stratification revealed persistently higher mortality in rural than urban areas, accompanied by a steeper decline in rural ASMR (Table 1; Figure 2C). In urban areas, ASMR decreased from 9.20 to 6.35 per 100,000 (AAPC = −2.42, 95% CI: −3.90% to −0.91). In rural areas, ASMR declined from 17.87 to 8.30 per 100,000 (AAPC = −6.05, 95% CI: −7.31% to −4.76). Trend comparability testing suggested that the decline trajectories differed significantly between urban and rural settings (*p* < 0.001; Supplementary Figure 1).

Across Eastern, Central, and Western China, ASMR declined significantly, although the magnitude and segmented patterns varied (Table 1; Figure 2D). During 2008–2012, Central China exhibited the highest ASMR, but from 2013 onward it became the lowest, while Eastern China remained relatively higher. In Eastern China, ASMR decreased from 14.11 to 8.06 per 100,000 (AAPC = −4.30, 95% CI: −6.42% to −2.14). In Central China, ASMR fell from 15.83 to 6.84 per 100,000 (AAPC = −6.16, 95% CI: −10.05% to −2.09), with the steepest drop occurring during 2010–2013. In Western China, ASMR declined from 13.15 to 7.90 per 100,000 (AAPC = −3.76, 95% CI: −4.52% to −2.98). Parallelism tests did not indicate significant differences in overall decline trends across the three regions (Supplementary Figure 1).

Notably, in several strata the reduction in CMR was smaller than that in ASMR (e.g., near-stable urban CMR despite declining ASMR), suggesting that shifts in population age structure—such as population ageing—may have partially offset the impact of declining risk on crude mortality. Accordingly, ASMR more accurately captures the underlying reduction in EC mortality risk over time (Table 1; Figure 2). Detailed Joinpoint outputs are shown in Supplementary Figure 2.

### 3.3 EC burden measured by PYLL and PYLLR

**Figure 3:**
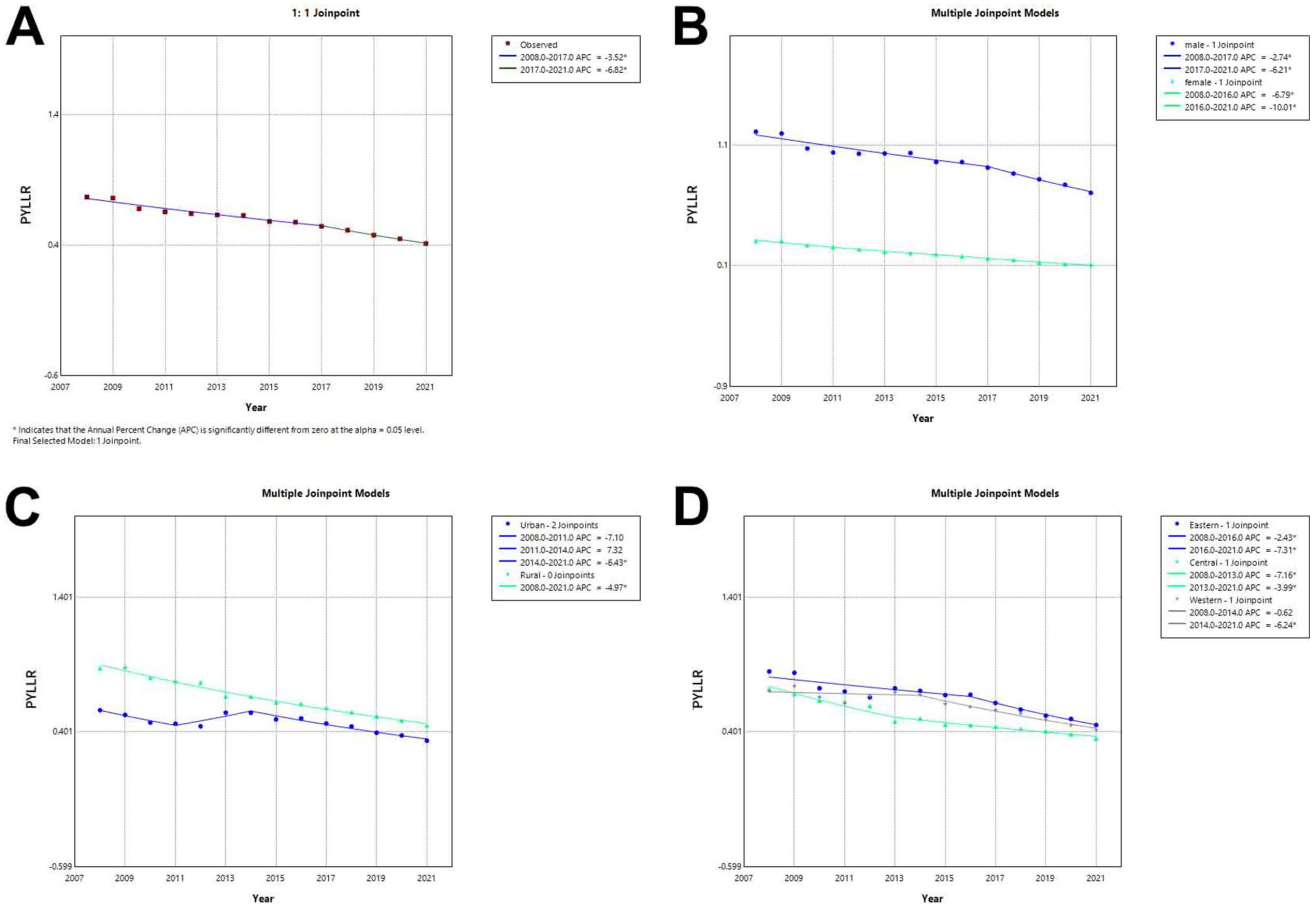
Joinpoint trends in PYLLR, 2008–2021. Annual potential years of life lost rate (PYLLR, ‰) in the overall population **(A)**, by sex **(B)**, by residence (urban/rural) **(C)**, and by region (eastern/central/western China) **(D)**. Points represent observed values, and solid lines represent fitted joinpoint segments. Segment-specific annual percent change (APC) estimates are shown in the inset; asterisks indicate APC values significantly different from zero.

From 2008 to 2021, the burden of premature mortality attributable to EC in China declined steadily (Table 2; Figure 3). Overall PYLLR decreased from 0.77‰ in 2008 to 0.41‰ in 2021. Joinpoint regression confirmed a statistically significant downward trend, with an AAPC of −4.55% (95% CI: −5.20 to −3.89%; Table 2). Segment-specific estimates suggested a faster decline in the later period (Figure 3A).

**Table 2:** PYLL and PYLLR of esophageal cancer from 2008 to 2021 in China.

Index		2008	2009	2010	2011	2012	2013	2014	2015	2016	2017	2018	2019	2020	2021	AAPC% (95%CI)	*t*	*p*
Total	PYLL	56645.00	56915.00	53343.00	50529.50	49342.00	142931.50	159038.50	149756.00	152194.50	146871.00	139267.50	131515.50	124521.00	109979.50			
	PYLLR	0.77	0.76	0.68	0.65	0.64	0.63	0.63	0.58	0.57	0.54	0.51	0.48	0.45	0.41	−4.55(−5.20, −3.89)	−13.27	<0.01
Male	PYLL	45650.50	45715.00	42983.50	40811.00	40386.00	119109.00	133500.00	125285.50	129070.00	125595.00	119402.50	114558.50	108970.50	95629.50			
	PYLLR	1.21	1.19	1.07	1.04	1.03	1.03	1.03	0.96	0.96	0.91	0.86	0.81	0.77	0.70	−3.82(−4.59, −3.05)	−9.56	<0.01
Female	PYLL	10994.50	11200.00	10359.50	9718.50	8956.00	23822.50	25538.50	24470.50	23124.50	21276.00	19865.00	16957.00	15550.50	14350.00			
	PYLLR	0.30	0.30	0.27	0.26	0.24	0.21	0.21	0.19	0.18	0.16	0.15	0.12	0.11	0.11	−8.04(−8.87, −7.21)	−18.24	<0.01
Urban areas	PYLL	14560.50	14068.50	14039.00	14275.00	13638.50	38269.50	44054.00	41074.50	44435.50	42499.00	41115.50	37360.00	36702.00	30873.00			
	PYLLR	0.56	0.53	0.47	0.46	0.44	0.54	0.54	0.50	0.50	0.46	0.44	0.40	0.38	0.34	−3.58(−6.94, −0.10)	−2.02	0.04
Rural areas	PYLL	42084.50	42846.50	39304.00	36254.50	35703.50	104662.00	114984.50	108681.50	107759.00	104372.00	98152.00	94155.50	87819.00	79106.50			
	PYLLR	0.88	0.89	0.80	0.78	0.77	0.67	0.67	0.62	0.61	0.58	0.55	0.52	0.49	0.45	−4.97(−5.36, −4.57)	−26.5	<0.01
Eastern China	PYLL	23985.00	24130.50	22339.00	21166.50	19773.50	63632.50	70409.00	66658.50	69233.50	65869.00	60788.50	57069.00	54490.50	48596.00			
	PYLLR	0.85	0.84	0.73	0.70	0.66	0.73	0.71	0.67	0.68	0.62	0.57	0.52	0.50	0.45	−4.34(−5.42, −3.25)	−7.66	<0.01
Central China	PYLL	18333.50	17641.50	16918.00	16977.50	16106.00	39599.50	45044.50	40684.50	42116.00	41835.00	40347.00	38712.50	36583.00	31715.50			
	PYLLR	0.72	0.68	0.63	0.63	0.59	0.48	0.50	0.46	0.45	0.44	0.43	0.41	0.38	0.35	−5.22(−6.07, −4.35)	−11.58	<0.01
Western China	PYLL	14326.50	15143.00	14086.00	12385.50	13462.50	39699.50	43585.00	42413.00	40845.00	39167.00	38132.00	35734.00	33447.50	29668.00			
	PYLLR	0.71	0.74	0.66	0.61	0.67	0.70	0.68	0.61	0.59	0.56	0.54	0.49	0.45	0.42	−3.68(−4.87, −2.49)	−5.96	<0.01

PYLL, potential years of life lost; PYLLR (‰), potential years of life lost rate; AAPC, average annual percent change; CI, confidence interval.

Sex-stratified analyses showed consistently higher PYLLR in males than in females, while both sexes experienced significant reductions over time (Table 2; Figure 3B). In males, PYLLR fell from 1.21‰ in 2008 to 0.70‰ in 2021 (AAPC = −3.82%; 95% CI: −4.59 to −3.05%). In females, PYLLR declined from 0.30‰ to 0.11‰ over the same years (AAPC = −8.04%; 95% CI: −8.87 to −7.21%), indicating a larger and faster reduction among females. The temporal trajectories differed significantly between males and females (*p* < 0.001; Supplementary Figure 1).

Across place of residence, rural areas consistently exhibited higher PYLLR than urban areas (Table 2; Figure 3C). Urban PYLLR decreased from 0.56‰ in 2008 to 0.34‰ in 2021 (AAPC = −3.58%; 95% CI: −6.94 to −0.10%), whereas rural PYLLR declined from 0.88‰ to 0.45‰ (AAPC = −4.97%; 95% CI: −5.36 to −4.57%). Although rural areas maintained a higher premature mortality burden throughout the study period, the overall downward trends did not differ significantly between urban and rural settings (*p* = 0.4287; Supplementary Figure 1).

Regional analyses indicated significant declines in PYLLR across Eastern, Central, and Western China, although the pace of reduction varied (Table 2; Figure 3D). In Eastern China, PYLLR declined from 0.85‰ in 2008 to 0.45‰ in 2021 (AAPC = −4.34%); in Central China, from 0.72‰ to 0.35‰ (AAPC = −5.22%); and in Western China, from 0.71‰ to 0.42‰ (AAPC = −3.68%). Overall, PYLLR levels were relatively higher in Eastern China. Trend comparability tests showed a statistically significant difference only between Central and Western China (*p* = 0.0398) (Supplementary Figure 1). Detailed Joinpoint outputs are shown in Supplementary Figure 2.

### 3.4 The age-period-cohort model analysis of EC

**Figure 4:**
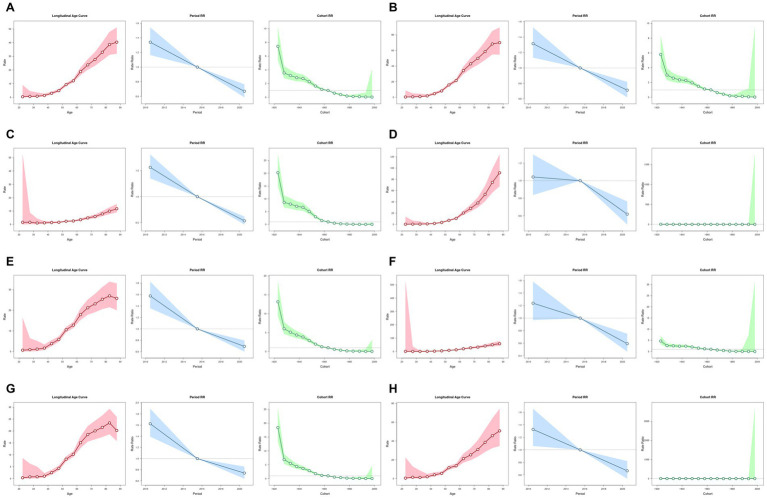
Age–period–cohort decomposition of esophageal cancer mortality. Age–period–cohort results for the total population **(A)**, males **(B)**, females **(C)**, urban populations **(D)**, rural populations **(E)**, eastern China **(F)**, central China **(G)**, and western China **(H)**. For each panel, the left plot shows the longitudinal age curve, the middle plot shows period relative risks (RRs), and the right plot shows cohort RRs. Shaded bands indicate 95% confidence intervals. Period and cohort RRs are shown relative to the reference categories defined by the NCI APC tool.

The age–period–cohort analysis indicated that EC mortality in China was jointly shaped by age, period, and birth-cohort effects (Figure 4). In the overall population, the longitudinal age curve increased progressively with advancing age, suggesting a strong positive age gradient in mortality risk that became markedly steeper after approximately 50 years. The period effect showed an overall decline in relative risk across calendar years, implying sustained period-related improvements after accounting for age and cohort influences. Regarding cohort effects, later birth cohorts generally exhibited lower relative risks than earlier cohorts, consistent with a generational reduction in mortality risk (Figure 4A). Notably, age–period–cohort patterns were not entirely uniform across subgroups, indicating heterogeneity in the underlying age-, period-, and cohort-related dynamics (Figure 4).

In sex-stratified analyses, both males and females demonstrated increasing risk with age; however, the age-associated rise was more pronounced among males during middle and older ages, reflected by a steeper age-curve slope (Figures 4B,C). Urban–rural comparisons suggested that the age-related escalation emerged earlier in rural populations, with the rural age curve showing a clearer upward inflection from around 40 years onward. Compared with the overall pattern, the period effect among urban residents varied less before 2015, and the cohort-effect curve appeared relatively flat, suggesting a more limited contribution of birth cohort to risk differentials in urban settings (Figures 4D,E). Regional stratification further showed stronger age effects in Central and Western China, and cohort effects also differed across regions. In particular, Central China displayed more evident changes across birth cohorts, indicating comparatively more pronounced intergenerational variation in mortality risk (Figures 4F–H).

However, the youngest and most recent birth cohorts were less precise, because of wider CI.

### 3.5 Age-specific mortality rate of EC

**Figure 5:**
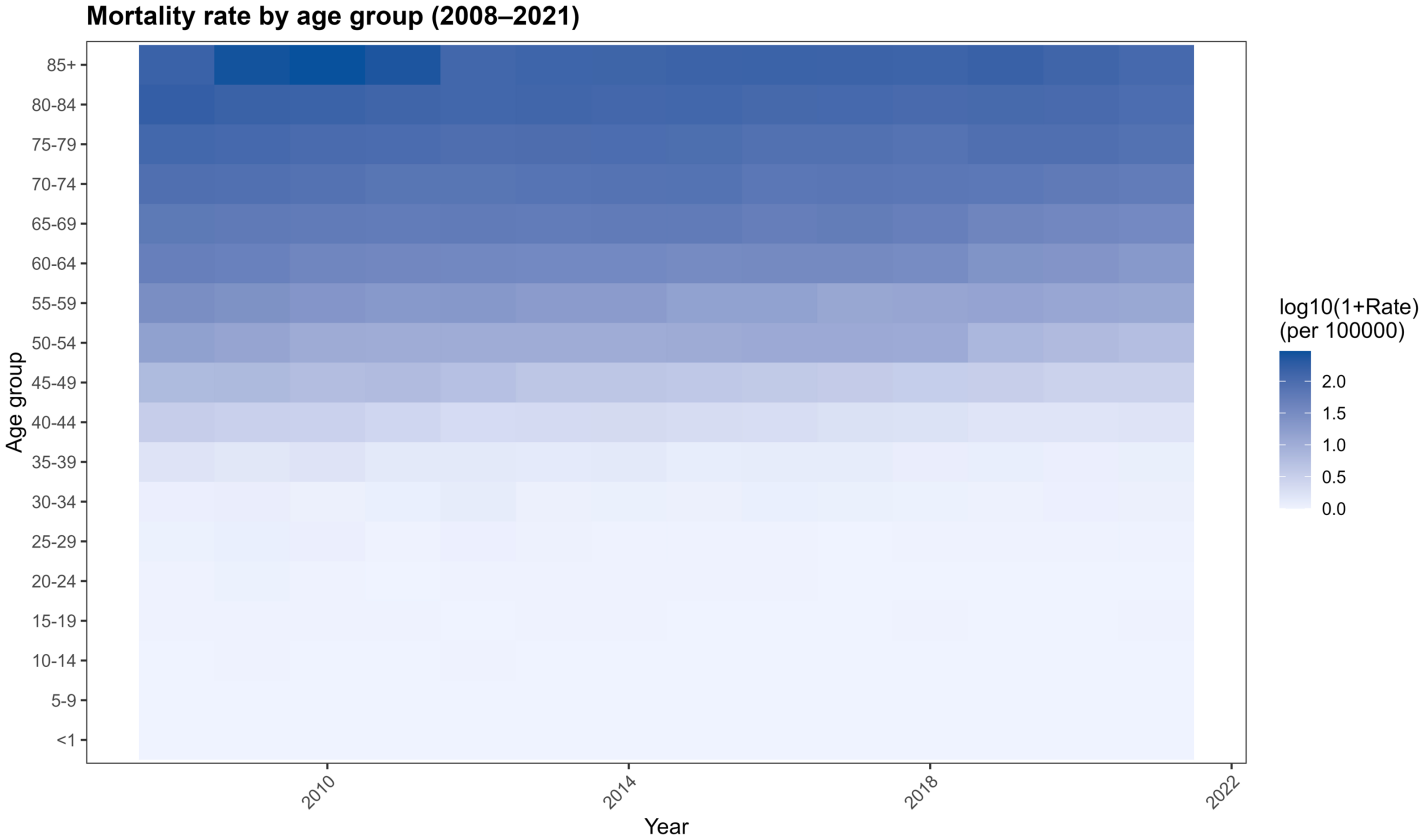
Heatmap of age-specific mortality rates, 2008–2021. Age-specific mortality rate (per 100,000) by calendar year and age group in the total population. Color intensity represents log10(1 + rate), with darker shading indicating higher mortality.

While the Joinpoint trend analysis and age–period–cohort models characterize the overall burden of EC in China and its temporal evolution across subgroups, they do not directly depict age-specific mortality patterns. Figure 5 therefore presents a heatmap of age-specific EC mortality rates in the total population from 2008 to 2021, with calendar year on the x-axis, age group on the y-axis, and color intensity representing the log-transformed mortality rate (log10[1 + rate per 100,000]).

Marked age dependence was evident. Mortality was close to zero in children, adolescents, and young adults (<30 years), corresponding to uniformly light shading. Color intensity increased substantially from approximately 45–54 years onward and remained high in older age groups, with the darkest shading observed among those aged ≥80 years—particularly the 85 + group—indicating the greatest mortality burden in the oldest age strata. Overall, the heatmap showed a progressive increase in mortality with age.

Across calendar time, the heatmap generally became lighter, most clearly among ages ~50–74, implying widespread declines in age-specific mortality over the study period. This pattern is consistent with the significant reduction in overall ASMR, indicating that the national decline was not solely attributable to demographic shifts or standardization procedures but was also reflected within several key age groups. Overall, Figure 5 visually reinforces the concentration of EC mortality in older adults and the broad decline in age-specific mortality over time.

### 3.6 Sexual differences in the burden of EC in different age groups

**Figure 6:**
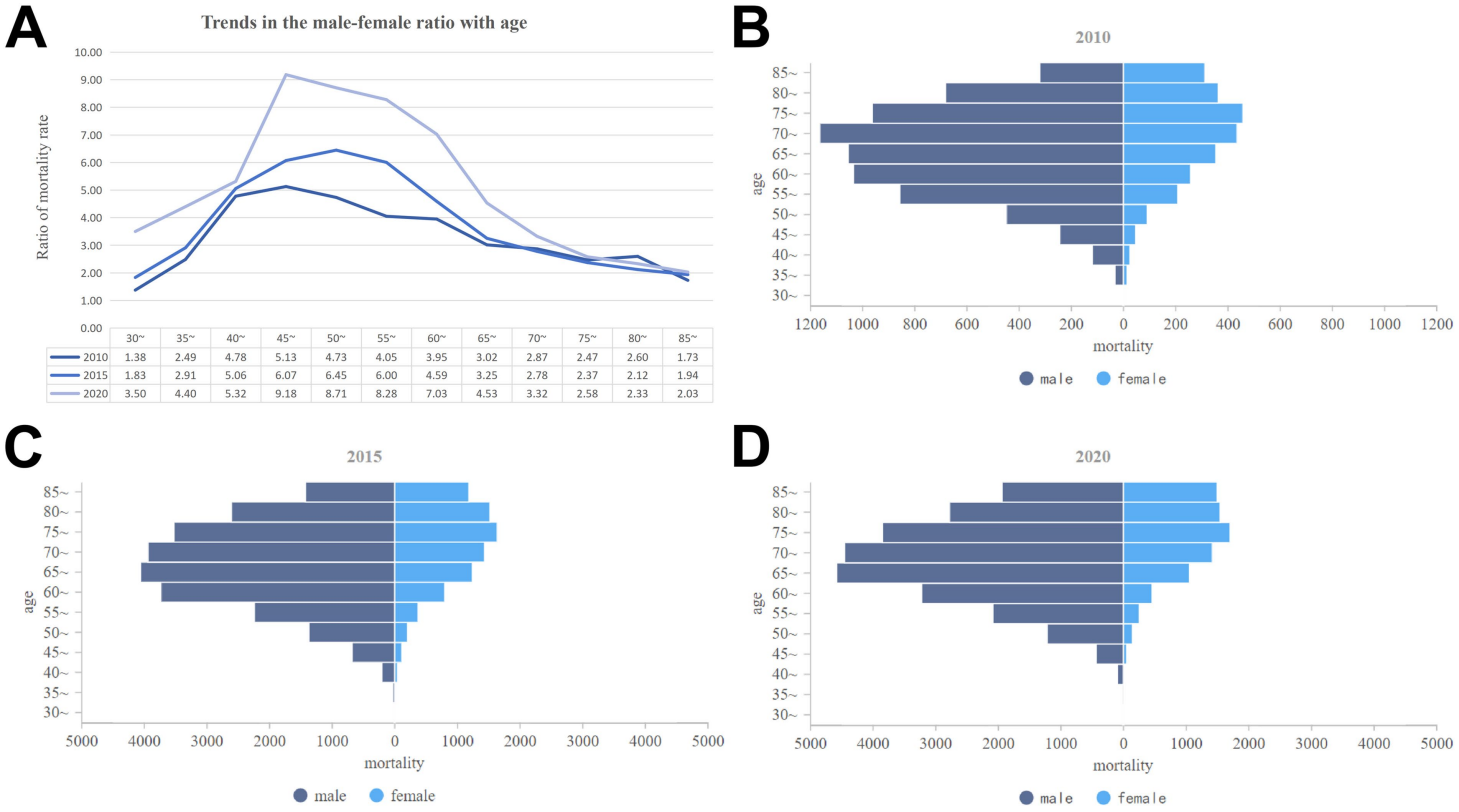
Sex differences in esophageal cancer mortality by age group. **(A)** Male-to-female mortality rate ratio (RR) by age group in 2010, 2015, and 2020. **(B–D)** Age-specific distributions of death counts by sex in 2010 **(B)**, 2015 **(C)**, and 2020 **(D)**, with males shown on the left and females on the right. These panels illustrate age-related sex differences in the distribution of mortality burden.

Given that EC mortality is close to zero before age 30, we restricted the age-specific sex-difference analysis to individuals aged ≥30 years (Figure 6). Figure 6A presents the male-to-female rate ratio (RR) of mortality by age group for 2010, 2015, and 2020. Overall, male mortality exceeded female mortality across all adult age groups (RR > 1), with the largest disparities observed in midlife and early older age. The RR increased after age 30, peaked broadly around 45–55 years, and then declined with advancing age. Among older adults (≥75 years), the RR decreased toward approximately 2, indicating attenuation of sex differences in late life. Compared with 2010 and 2015, RRs in 2020 were generally higher in midlife, suggesting a more pronounced excess male risk in recent years (Figure 6A).

Figures 6B–D further depict the sex-specific distribution of EC deaths (counts) across age groups in 2010, 2015, and 2020. Male deaths peaked at ages 60–70, whereas the peak for females occurred later, predominantly at ages 75–80 (Figures 6B–D). Importantly, the age at which death counts peak reflects not only risk but also the underlying population size and age structure; thus, these count-based results primarily characterize the distribution and concentration of burden across the age spectrum (Figures 6B–D).

### 3.7 Projected trends in ASMR for the next 15 years

**Figure 7:**
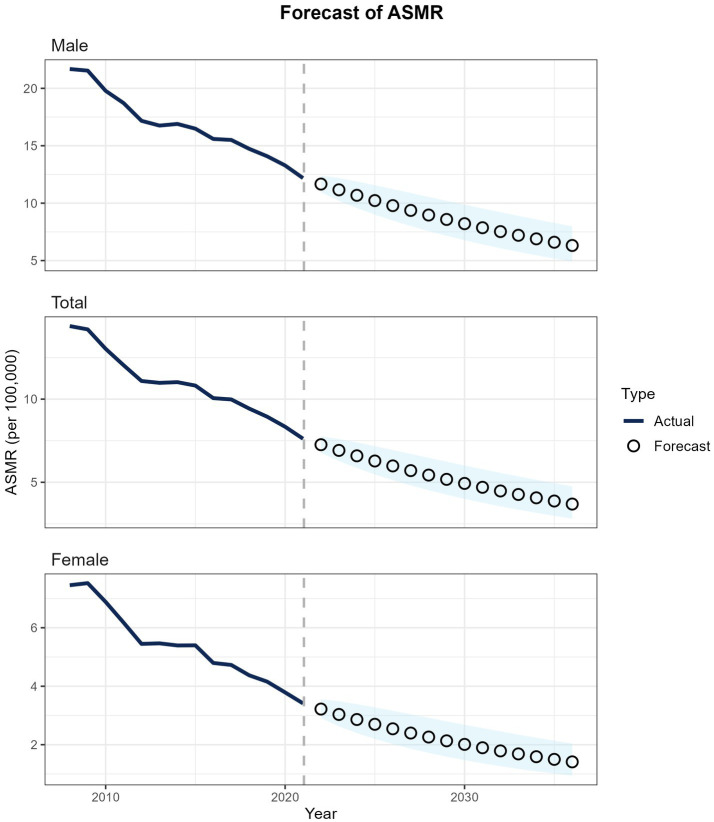
ARIMA-based projections of ASMR for 2022–2036. Observed ASMR (solid line) during 2008–2021 and projected ASMR (open circles) during 2022–2036 for males, the total population, and females. Shaded bands indicate 95% prediction intervals. The vertical dashed line marks the transition from observed to projected values.

Using annual ASMRs for EC in China from 2008 to 2021, we fitted ARIMA time-series models—implemented on log/Box–Cox–transformed rates and back-transformed to the original scale—to project ASMRs for 2022–2036 (Figure 7). In Figure 7, solid lines represent observed values, open circles denote point forecasts, and shaded bands indicate the 95% prediction intervals (95% PI). Overall, the projections suggest that EC ASMR will continue to decline over the next 15 years. The national ASMR was 7.62 per 100,000 in 2021 and is expected to decrease steadily to 3.69 per 100,000 by 2036 (95% PI: 2.81–4.75), corresponding to an estimated 51.6% reduction relative to 2021, indicating a substantial further decrease in population-level mortality risk (Figure 7).

Sex-stratified forecasts showed persistently higher ASMRs in males than in females throughout the projection period, while both sexes exhibited sustained downward trends (Figure 7). Among males, ASMR is projected to decline from 12.19 per 100,000 in 2021 to 6.31 per 100,000 in 2036 (95% PI: 4.92–7.99). Among females, ASMR is projected to decrease from 3.42 per 100,000 in 2021 to 1.41 per 100,000 in 2036 (95% PI: 0.94–2.04). The relative reduction is larger in females (58.7%) than in males (48.2%); however, because male rates start from a higher baseline, the absolute ASMR is expected to remain markedly higher in males, suggesting that sex disparities may persist in the coming years (Figure 7). Notably, PIs widen over time across all panels, indicating increasing uncertainty for longer-horizon forecasts, particularly beyond 2030.

## 4 Discussion

Using data from DSP system, this study provides a comprehensive picture of the changing burden of EC in China from 2008 to 2021 and its likely future trajectory. Overall, both mortality risk and premature mortality burden declined substantially during the study period, indicating a broad improvement in disease burden at the population level. At the same time, these improvements were not entirely uniform: the patterns of change in PYLLR did not fully parallel those of ASMR, suggesting that reductions in mortality risk and changes in premature mortality may be influenced by partly different factors. The age–period–cohort results further support that these trends were shaped not only by ageing-related risk accumulation, but also by broader temporal improvements and generational shifts in risk. Looking ahead, the observed downward trend in ASMR may continue, although uncertainty increases over longer time horizons. Despite the overall improvement, important disparities persisted, with consistently higher mortality in males and rural residents, while regional differences narrowed over time and the relative ranking of regions changed.

We observed a sustained and statistically significant decline in EC ASMR in China from 2008 to 2021. The age–period–cohort analysis further revealed a clear pattern consistent with a “period improvement,” suggesting that, beyond population ageing and cohort-related differences, broader time-varying forces may have shifted the overall risk downward during the study period. Prior evidence indicates that EC is associated with multiple modifiable factors, including tobacco use, alcohol consumption (21), unhealthy diet and poor nutritional status (22), eating behaviors (23), oral hygiene, infections (24), and environmental exposures (25). At the macro level, a higher Socio-demographic Index has been linked to lower age-standardized mortality (26). Within this context, China’s sustained efforts since the early 2000s to implement screening and early detection–treatment programs in high-incidence areas may have been an important contributor to the observed period-related improvement (27).

China’s National Cancer Prevention and Control Plan (2004–2010) designated EC as a priority target for control, which helped sustain programmatic investment and strengthen cancer registration and surveillance infrastructure (28). Public reports indicate that, by 2018, more than 2.16 million individuals had undergone upper gastrointestinal endoscopic screening, with the early detection rate for malignant lesions exceeding 70% (27). In parallel, sustained research efforts—led by both international groups (e.g., NCI, IARC) and domestic teams—have been conducted in high-incidence areas, generating evidence to inform prevention and control strategies (29, 30). Collectively, these government-led policies, continued fiscal support, and ongoing research initiatives may have contributed to the observed decline in mortality risk during the study period. Notably, the decrease in the CMR was substantially smaller than that in ASMR (with CMR remaining near a plateau in some strata despite continued declines in ASMR), suggesting that shifts in population age structure—particularly population ageing—may have partially offset the impact of declining underlying risk on crude mortality.

Prior evidence from both global and Chinese studies consistently indicates substantial heterogeneity in EC mortality burden, with particularly pronounced inequalities by sex, urban–rural residence, and geographic region (11, 30, 31). In our analysis, male ASMR remained persistently higher than female ASMR throughout the study period, and the male-to-female mortality RR was generally >2 among individuals aged ≥30 years. This disparity may partly reflect higher male exposure to key risk factors, such as tobacco use, heavy alcohol consumption, and other behavior-related determinants (32). Studies have reported that tobacco and alcohol use are the primary drivers of the burden of mortality and disability in men, whereas in women, high BMI is the predominant factor (33). However, lifestyle factors alone are unlikely to fully account for the sex gap. Previous studies have reported better EC–specific survival and overall survival in women than in men, and have proposed potential protective effects of sex hormones, with the greatest sex differences observed around the perimenopausal period and attenuation thereafter (34, 35). Consistent with these observations, we found that the RR peaked in midlife (with the highest RR in 2020 occurring at approximately 45–55 years, reaching ~9.18) and diminished at older ages. Moreover, the peak in female death counts was shifted to older age groups relative to males (predominantly 75–80 years in women vs. 60–70 years in men), aligning with prior reports. Importantly, our data do not allow separation of incidence-driven from survival-driven contributions to sex differences in mortality; further studies incorporating histologic subtype, stage distribution, and treatment information are needed to clarify underlying mechanisms.

With respect to the urban–rural gradient, rural populations consistently exhibited higher ASMR and PYLLR than urban populations throughout the study period; however, ASMR declined more rapidly in rural areas (AAPC ≈ −6.05%) than in urban areas (AAPC ≈ −2.42%), indicating that the mortality burden remained disproportionately concentrated in rural settings. First, long-standing disadvantages in rural communities—such as low per capita GDP and low educational attainment—are significantly associated with higher rates of morbidity and mortality (36, 37). Rural residents also tend to have higher prevalences of smoking, heavy alcohol use, and unhealthy dietary practices (14). In certain high-risk rural belts (e.g., the Taihang Mountain region and surrounding areas), exposure to environmental carcinogens may further contribute to excess risk (38). These differences in lifestyle and environmental exposures have been shown to significantly increase the risk of EC (39–41). As economic development, urbanization, and targeted screening programs in high-incidence areas expanded, the decline in mortality risk may have been more pronounced in rural populations, contributing to partial convergence in some metrics. Notably, the trend-comparability test did not detect a statistically significant difference in the declining trajectories of urban versus rural PYLLR, underscoring that rural populations remain a priority for prevention efforts and resource allocation.

At the regional level, ASMR declined significantly in eastern, central, and western China, but the relative ordering changed over time. The central region, which initially had the highest levels, became the lowest by the end of the study period, whereas the eastern region showed the highest ASMR in 2021; overall, interregional differences narrowed, suggesting convergence. Historically, China’s EC “hotspot” has been concentrated along the Taihang Mountain–Huai River corridor spanning parts of central and eastern China (13). Prominent regional environmental and dietary risks are independent drivers of the exceptionally high incidence of EC (10); genetic susceptibility has also been proposed as a contributor (42), although its causal importance in shaping regional patterns remains to be established. We speculate that the steeper decline observed in the central region may be partly attributable to sustained investment and expanded coverage of early detection and early treatment programs in selected high-incidence counties (43). However, because screening resources and program sites are unevenly distributed, other areas may have benefited less (42), potentially contributing to the observed shifts in regional rankings. Despite substantial national declines, some high-risk counties still experience incidence and mortality far above the national average (44), indicating that the traditional high-burden belt remains a priority and warrants continued, locally tailored comprehensive prevention and control. Finally, improvements over time in surveillance completeness and cause-of-death coding may have influenced regional comparisons and trend estimates; although the DSP system applies multi-level quality assurance, we cannot fully exclude the possibility that diagnostic and attribution enhancements contributed to part of the observed differences.

The age–period–cohort analyses provide a structural interpretation of changes in EC mortality risk across age, calendar time, and birth cohorts. Overall, the age effect displayed a clear monotonic gradient, consistent with the pattern in Figure 5 showing a marked intensification after approximately 50 years of age and persistently high rates in older groups. This likely reflects the combined impact of cumulative lifetime exposures and aging-related biological processes, together with a higher burden of comorbidities and reduced treatment tolerance in later life, which concentrates mortality burden in the oldest age strata (45). Notably, the age curve was flatter among women, which may relate not only to longstanding sex differences in exposure profiles for key risk factors (46) but also to biological determinants (47); these hypotheses require validation using data on histology, stage distribution, and treatment.

The period effect suggested a broad improvement over the study period, which may partly reflect macro-level changes accompanying rapid urbanization. In high-incidence areas of China, nutritional status and drinking-water conditions have improved substantially over the past decade (48). In addition, since 2009, China has continuously implemented upper gastrointestinal cancer screening programs for high-risk populations. For example, nearly 90,000 individuals underwent endoscopic screening over 5 years in 12 high-incidence counties and cities in Henan Province, with an upper gastrointestinal cancer detection rate of 2.34%; notably, 81.7% of detected cases were early-stage cancers (49), supporting the feasibility and effectiveness of endoscopic screening and minimally invasive early treatment in high-risk adults aged 40–69 years. Meanwhile, major advances in esophageal cancer surgery and multidisciplinary management have likely also contributed to improved outcomes (50). By contrast, cohort effects varied across subgroups. In urban and western populations, the cohort curves were relatively flat. This heterogeneity may reflect differences in long-term exposure profiles within traditional high-risk areas, the geographic concentration of screening and prevention resources, and distinct trajectories of lifestyle and environmental exposures across urban–rural and regional settings.

Using annual data from 2008 to 2021, we fitted an ARIMA time-series model and extrapolated EC ASMRs for 2022–2036. Under an assumption that historical patterns persist, the projections indicate continued declines in ASMR for both men and women; however, male ASMR is expected to remain consistently higher, suggesting that sex-related disparities in mortality burden are likely to persist in the near to mid term. Importantly, in the context of ongoing population aging, reductions in risk may not translate into proportional decreases in the absolute number of deaths, and the overall death burden may remain elevated or decline only modestly. However, these estimates are best interpreted as data-driven extrapolations rather than deterministic predictions, because future trajectories may be altered by changes in screening coverage, diagnostic practice, treatment access, population ageing, or other structural shifts.

Unfortunately, the DSP dataset does not include individual-level risk factors or clinical information (e.g., BMI, alcohol intake, smoking prevalence and intensity). Our study was an ecological analysis based on aggregated surveillance data. Accordingly, the observed differences across sex, residence, and region should not be interpreted as individual-level causal associations. These subgroup contrasts may also be confounded by unmeasured variation in smoking, alcohol consumption, diet, socioeconomic status, screening uptake, stage at diagnosis, and treatment access. To gauge the potential impact of this limitation, we consulted published evidence on smoking, alcohol use, high-risk dietary exposures, and socioeconomic development and conducted a preliminary comparison with patterns observed in our dataset (38); however, this approach cannot substitute for formal individual-level attribution analyses.

Second, cause of death was classified using ICD-10 code C15 without further stratification by histological subtype (e.g., squamous cell carcinoma vs. adenocarcinoma). Given the distinct epidemiology and risk-factor profiles across subtypes (51), this may constrain mechanistic interpretation and etiologic inference. Finally, because data were only available through 2021, we were unable to evaluate potential post–COVID-19 changes in EC mortality related to disruptions in healthcare utilization. Future studies should integrate longer time series, individual-level information, and external data on exposures and screening coverage to enable more informative mechanistic testing and scenario-based assessments.

## 5 Conclusion

Between 2008 and 2021, the mortality burden of esophageal cancer in China declined substantially, but marked disparities by sex, urban–rural residence, and region persisted. In addition, the declining patterns of mortality risk and premature mortality were not fully concordant, suggesting that they may be influenced by partly distinct factors. Age–period–cohort analysis further indicated that the contributions of period-related improvements and intergenerational changes were not uniform across population subgroups. Therefore, future prevention and control efforts should move beyond reducing overall risk alone and prioritize middle-aged and older men, rural residents, and historically high-risk areas. Key strategies include strengthening risk-factor prevention, expanding risk-stratified screening and early detection, and improving timely diagnosis and treatment. Given persistent inequalities in healthcare resource distribution, it is also necessary to enhance health education in underserved and remote areas and to strengthen the capacity of primary healthcare institutions for case recognition, referral, pathology, and endoscopy in traditional high-burden belts and other resource-limited regions. Such targeted strategies will be essential to sustain the downward trend in esophageal cancer burden and to reduce persistent health inequalities in China.

## Data Availability

The original contributions presented in the study are included in the article/Supplementary material, further inquiries can be directed to the corresponding authors.
