# Supplementary material for: Esophageal cancer mortality in China, 2008–2021: trends, disparities, and projections
Source: Front Public Health. 2026 May 25;14:1795219. doi: 10.3389/fpubh.2026.1795219 (PMC13243382; doi:10.3389/fpubh.2026.1795219)
Supplement: Supplementary file 1 [file Supplementary_file_1.docx]

The calculation is as follows:

1. The crude mortality rate (CMR) was calculated as the number of deaths divided by the population and multiplied by 100,000 (per 100,000 population).

2. The calculation of the Age-standardized mortality rate (ASMR) is as follows:

$$\mathrm{ASMR}=\frac{\sum nP_{x}*nM_{x}}{\sum nP_{x}}$$

In the formula, $nP_{x}$denotes the age-specific population in the standard population, $nM_{x}$denotes the age-specific mortality rate in the population to be standardized, $n$denotes the width of each age group, and $x$denotes the starting age of each age group.

3. PYLL = ai · di (where di is the average age of the i-th age group; ai is the residual age, calculated as ai = 70 - xi + 0.5, with xi being the average age of the i-th age group. The addition of 0.5 is applied to eliminate the influence of nominal age counting).

4. The PYLLR (in ‰) = PYLL · 1000 / N (where N is the total number of people aged 1 to 70 in the surveyed population).
