# Supplementary figures and images for: Esophageal cancer mortality in China, 2008–2021: trends, disparities, and projections
Source: Front Public Health. 2026 May 25;14:1795219. doi: 10.3389/fpubh.2026.1795219 (PMC13243382; doi:10.3389/fpubh.2026.1795219)

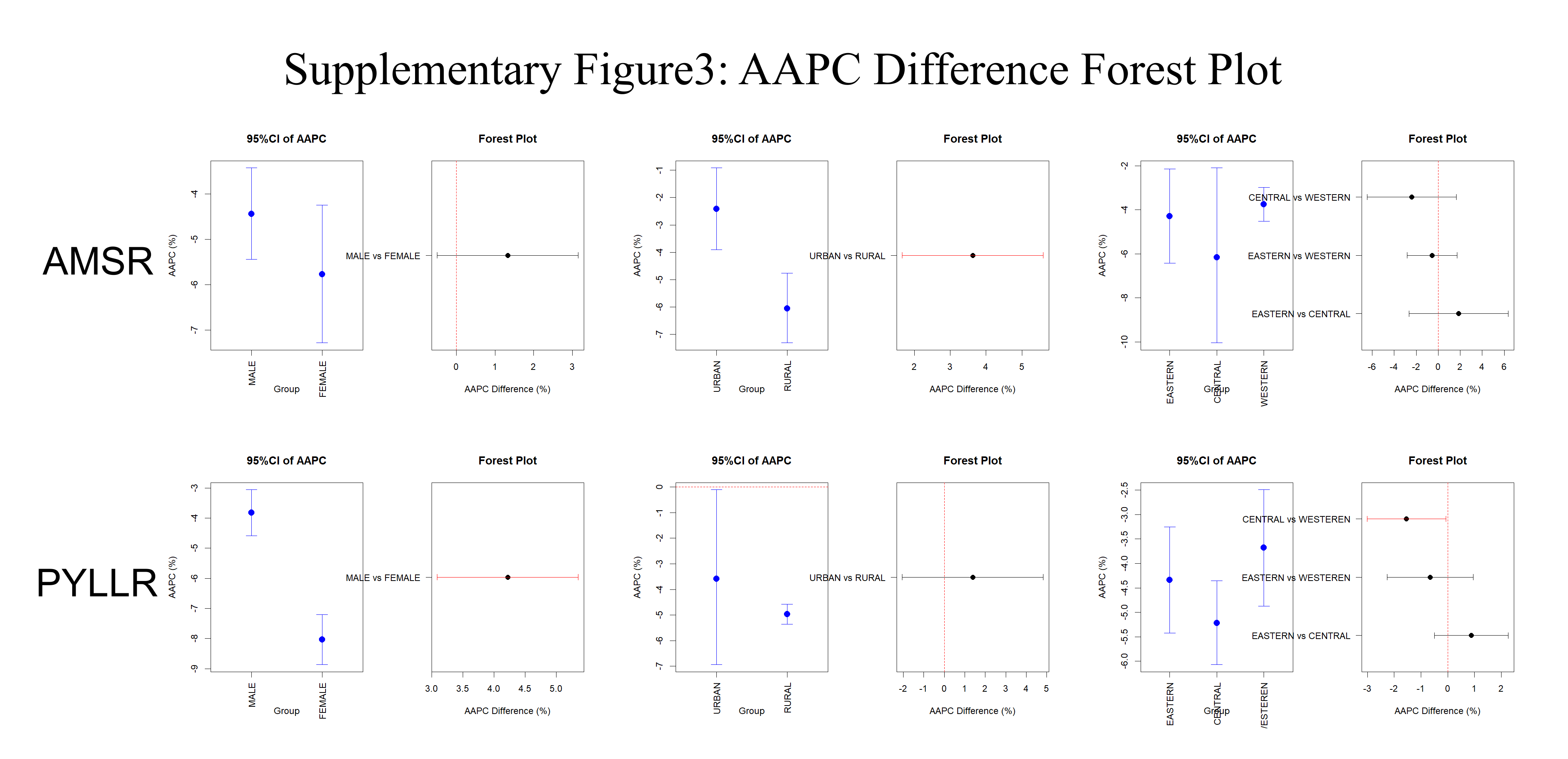

Supplement: Supplementary file 2 [file Image_1.tif]

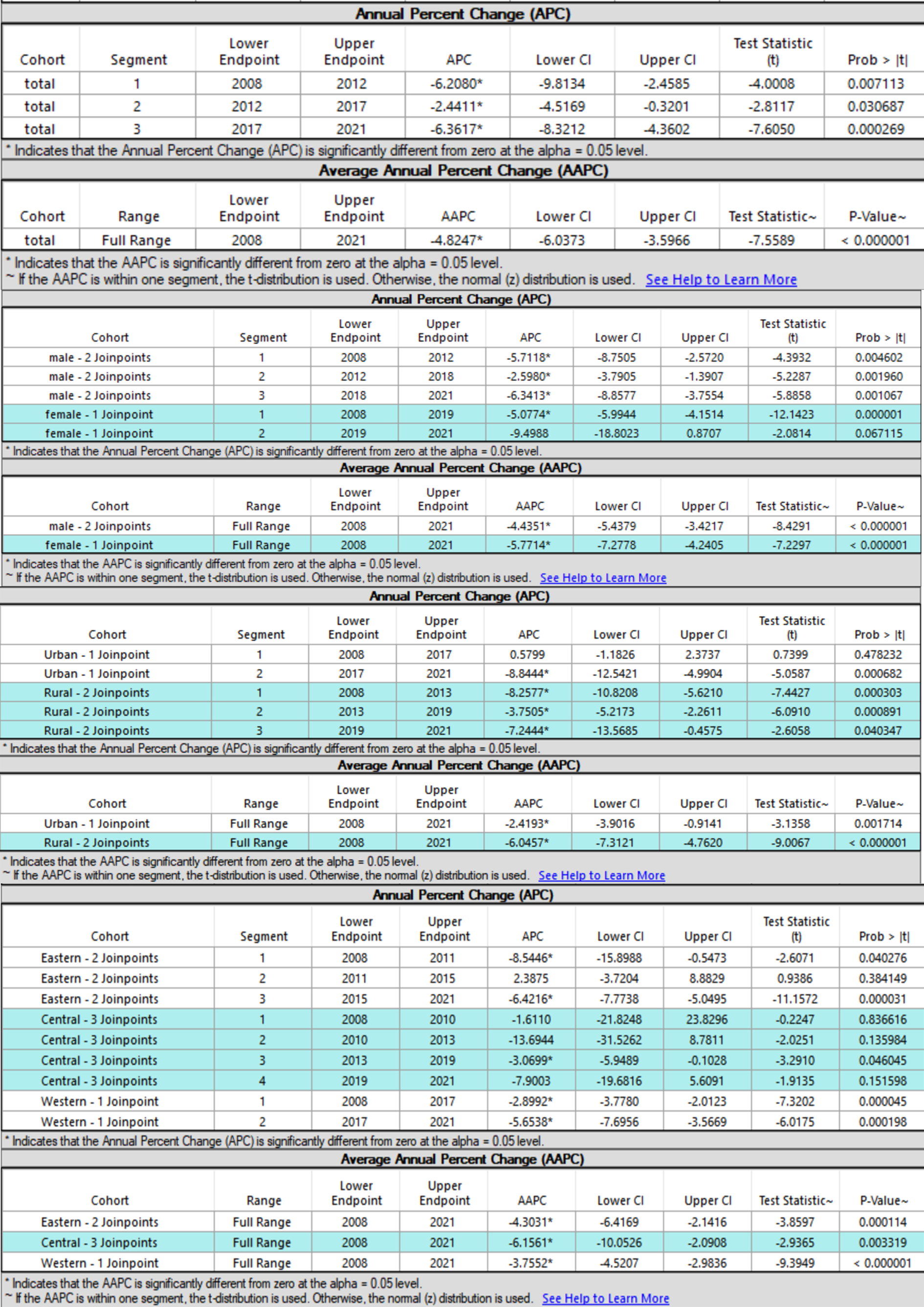

Supplement: Supplementary file 3 [file Image_2.tif]

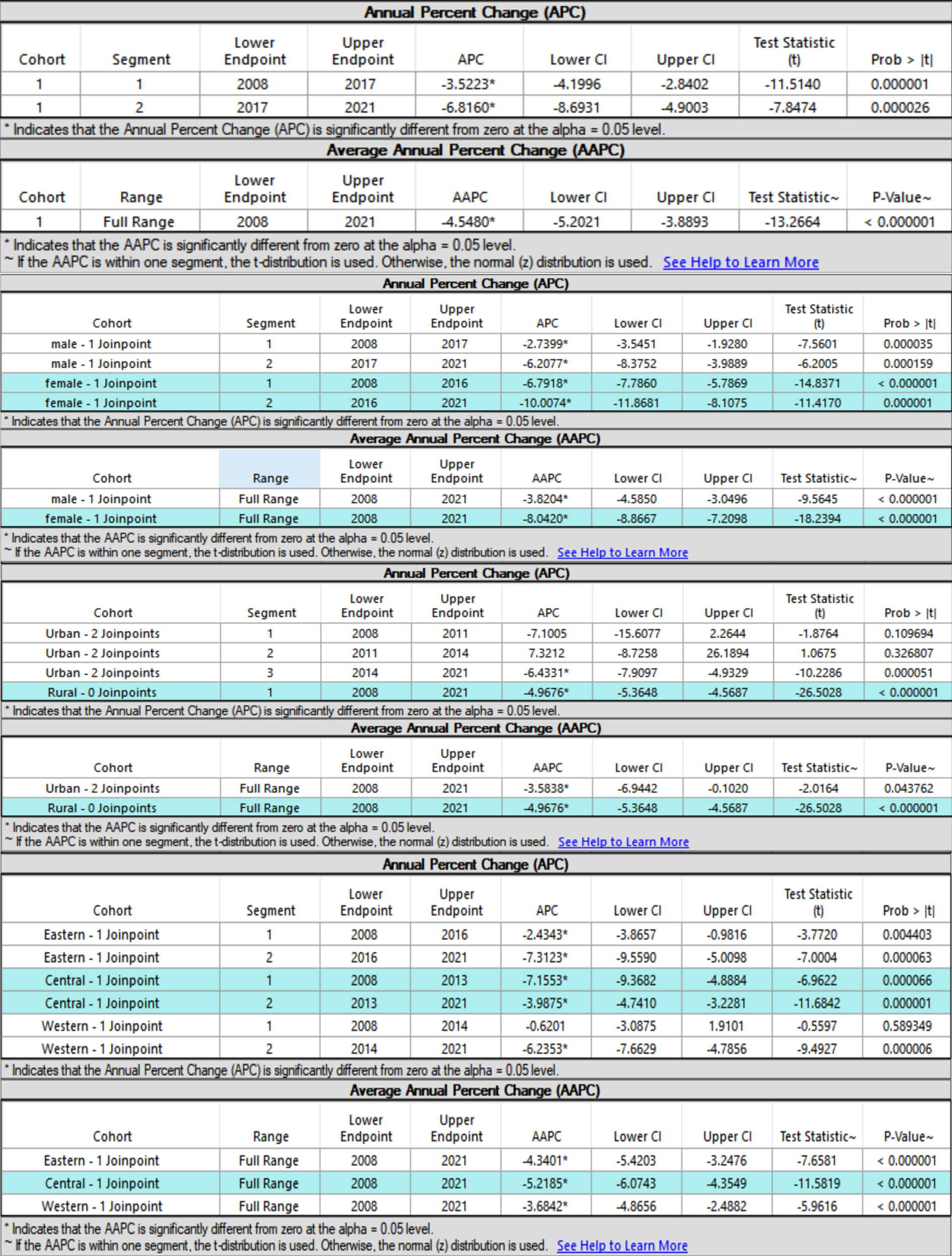

Supplement: Supplementary file 4 [file Image_3.tif]
